# Selenium intakes and plasma selenium of New Zealand toddlers: secondary analysis of a randomised controlled trial

**DOI:** 10.1017/S0007114522002379

**Published:** 2023-04-14

**Authors:** Lisa Daniels, Jillian J. Haszard, Rosalind S. Gibson, Rachael W. Taylor, Elizabeth A. Fleming, Jody C. Miller, Christine D. Thomson, Anne-Louise M. Heath

**Affiliations:** 1 Department of Medicine, University of Otago, PO Box 56, Dunedin 9054, New Zealand; 2 Department of Human Nutrition, University of Otago, PO Box 56, Dunedin 9054, New Zealand; 3 Biostatistics Centre, University of Otago, PO Box 56, Dunedin 9054, New Zealand

**Keywords:** Selenium intake, Plasma selenium, Toddlers, New Zealand

## Abstract

Little is known about Se intakes and status in very young New Zealand children. However, Se intakes below recommendations and lower Se status compared with international studies have been reported in New Zealand (particularly South Island) adults. The Baby-Led Introduction to SolidS (BLISS) randomised controlled trial compared a modified version of baby-led weaning (infants feed themselves rather than being spoon-fed), with traditional spoon-feeding (Control). Weighed 3-d diet records were collected and plasma Se concentration measured using inductively coupled plasma mass spectrometry (ICP-MS). In total, 101 (BLISS *n* 50, Control *n* 51) 12-month-old toddlers provided complete data. The OR of Se intakes below the estimated average requirement (EAR) was no different between BLISS and Control (OR: 0·89; 95 % CI 0·39, 2·03), and there was no difference in mean plasma Se concentration between groups (0·04 μmol/l; 95 % CI −0·03, 0·11). In an adjusted model, consuming breast milk was associated with lower plasma Se concentrations (–0·12 μmol/l; 95 % CI −0·19, −0·04). Of the food groups other than infant milk (breast milk or infant formula), ‘breads and cereals’ contributed the most to Se intakes (12 % of intake). In conclusion, Se intakes and plasma Se concentrations of 12-month-old New Zealand toddlers were no different between those who had followed a baby-led approach to complementary feeding and those who followed traditional spoon-feeding. However, more than half of toddlers had Se intakes below the EAR.

Se is a trace element that has been reported to play an important role in many areas of the human body including thyroid metabolism, cognitive function, immune function and growth^(1–3)^. While there are many functions of Se in the human body, there appears to be a U-shaped curve of adverse effects, where deficits and excessive Se concentrations can impact on the risk and onset of certain diseases^(4,5)^.

It is well known that groups of the New Zealand population, particularly in the South Island, are not meeting the recommended intakes of Se and have lower status than what has been reported in some other countries^(6,7)^. This is in most part due to low Se concentrations in the soil^(6,8)^. However, only one study has reported information on Se intakes and status of South Island (New Zealand) infants and toddlers, in 1998–1999^(7)^. Also, it is not known whether the increasingly popular alternative approach to introducing solid foods to infants known as Baby-Led Weaning (in which infants feed themselves rather than being spoon-fed) influences Se intakes. A baby-led approach to infant feeding has been shown to influence food and nutrient intake in infancy^(9–11)^ so may influence Se intake and therefore status.

The objectives of this study were to determine: (1) whether Se intakes and plasma Se concentrations differ between 12-month-old New Zealand toddlers who followed a baby-led approach to complementary feeding and those who followed traditional spoon-feeding, (2) what food sources contribute to Se intakes of 12-month-old toddlers and (3) what factors are associated with plasma Se concentrations in New Zealand 12-month-old toddlers.

## Experimental methods

This is a secondary analysis of the Baby-Led Introduction to SolidS (BLISS) study which has been described in full elsewhere^(12)^. The information provided here is information of relevance to the current analysis. The BLISS randomised controlled trial investigated the impact of a modified version of Baby-Led Weaning on infant outcomes including growth^(13)^, choking^(14)^, Fe^(15)^ and Zn^(16)^. Women (*n* 206) were recruited (between November 2012 and March 2014) during the third trimester of their pregnancy through an opt-out enrolment process (i.e. all potentially eligible women were approached and offered the opportunity to participate) at the only birthing facility in the city of Dunedin, New Zealand (southern region of New Zealand). Participants were eligible if they: spoke English or Te Reo Māori (indigenous language of New Zealand); planned to live in the area of Dunedin, New Zealand, until their child was at least 2 years of age and were 16 years of age or older. Exclusion criteria were if the infant was born before 37 weeks gestation or had a congenital abnormality, physical condition or intellectual disability that was likely to affect their feeding or growth. The study was conducted in accordance with the Declaration of Helsinki for research involving human subjects and was approved by the New Zealand Lower South Regional Ethics Committee (LRS/11/09/037). Adult participants gave written informed consent.

**Fig. 1.**
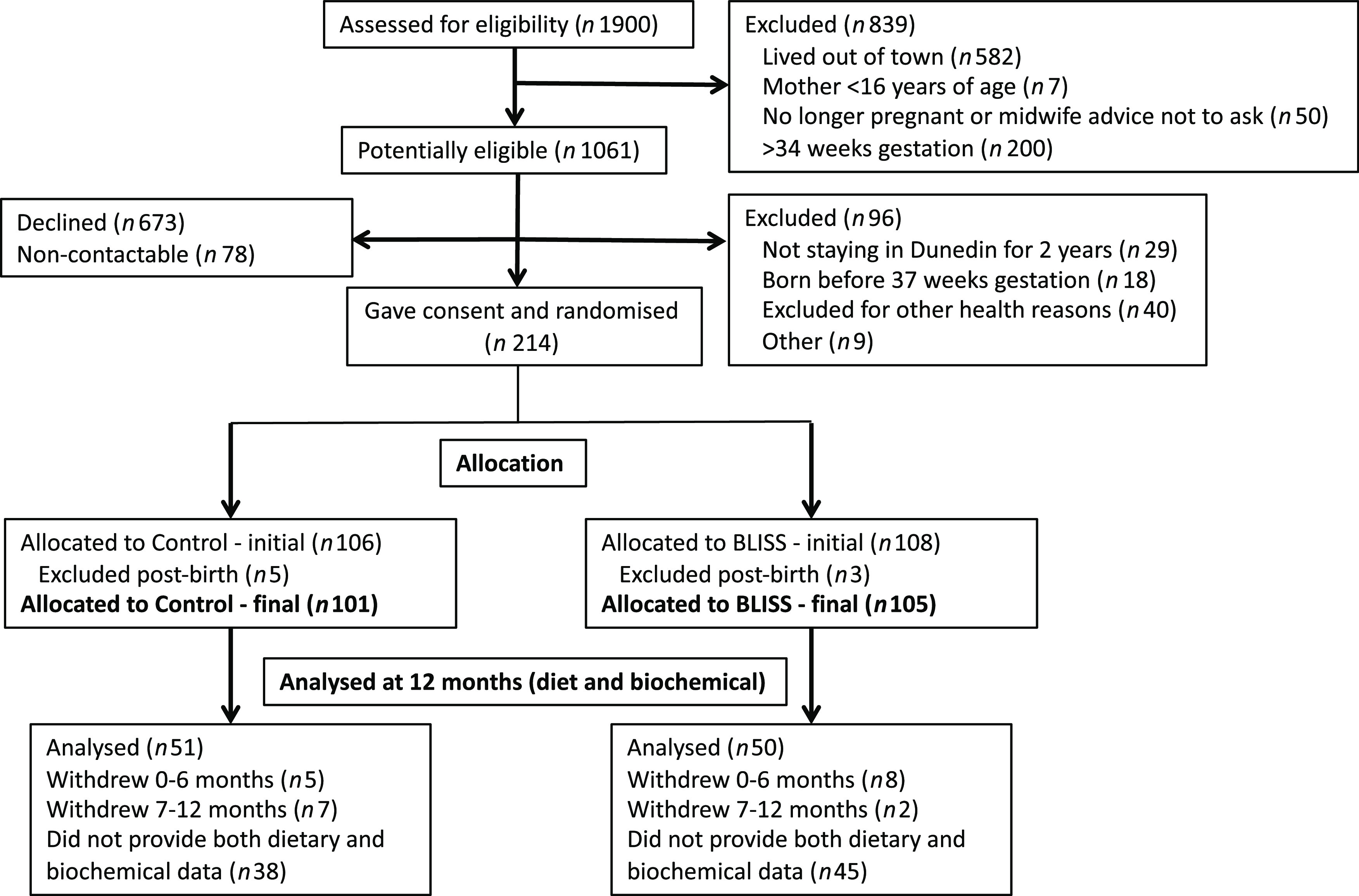
Flow diagram of participants through the BLISS study, with an emphasis on the participants analysed for this secondary analysis. BLISS, Baby-Led Introduction to SolidS.

After obtaining consent, participants were randomised into the Control or BLISS intervention group (Fig. 1). Both groups received standard ‘Well Child’ care available to all New Zealand children up to 5 years of age^(17)^, and BLISS participants received further education and support regarding the BLISS approach (i.e. infant self-feeding, with no spoon-feeding, from 6 months of age with modifications to address concerns about the possible risks of Fe deficiency, choking and growth faltering^(18)^).

Demographic data were collected by questionnaire, including the participant’s address which was used to determine the level of household deprivation with the New Zealand Index of Deprivation (NZDep) score^(19)^. Infant sex, birth weight and gestational age at birth were obtained from hospital records. When the child was 12 months of age, parents were asked about the mother’s smoking status during pregnancy (daily, occasional, quit during pregnancy, non-smoker).

Parent participants completed a weighed diet record on three randomly assigned non-consecutive days (two week days and one weekend day), using dietary scales (Salter Electronic, Salter Housewares Ltd.) accurate to ±1 g, when the child was aged 12 months. Parents recorded all information on what their child ate and drank (time of day the food was consumed, type and brand of food, cooking method), the total food weight before offering and at the end of the eating occasion (i.e. that was leftover), any dietary supplements taken and any recipes used. Data from the 3-d weighed diet record were analysed using the database Kai-culator (Version 1.13s, University of Otago) which contains data from the New Zealand Food Composition Database^(20)^, recipes from the 2008/2009 New Zealand Adult Nutrition Survey^(21)^, and commercial infant foods collated by the research team^(22)^. During the dietary data entry, food items were selected carefully to ensure that they contained appropriate Se concentrations, notably for flour and bread products because of the known regional variations in their Se content^(6)^. For toddlers consuming breast milk (no infant formula), intakes were estimated to be 448 g/d^(23)^. If the toddler was mixed fed (breast milk and infant formula), the amount of breast milk was estimated to be the amount left after subtracting their weighed consumption of infant formula (g per day) from 448 g/d. The Se content of breast milk was assumed to be 2 μg/100 g^(20)^. The data from the 3-d weighed diet record were used to determine daily intakes of: energy, protein, Se and Se intakes from breast milk, infant formula and nine food groups (‘seafood’, ‘breads and cereals’, ‘meat’, ‘dairy’, ‘fruits and vegetables’, ‘eggs’, ‘nuts and seeds’, ‘legumes’ and ‘miscellaneous’). Intakes of energy, protein and Se were then entered into the Multiple Source Method programme^(24)^ to calculate ‘usual’ daily intakes, which accounts for intra-individual variation in intake.

A non-fasting venous blood sample was obtained at 12 months of age to determine plasma Se, C-reactive protein and α_1_-acid glycoprotein concentrations, as well as for determination of biomarkers of Fe and Zn status, as has been reported earlier^(16,17)^. For the purposes of Zn analysis, parents were instructed to give their child a milk feed 90 min prior to blood collection and then no other food or drink until after the blood test, and a rigorous trace-element free protocol was used during both blood collection and analysis (including use of trace-element free lithium heparin anticoagulated S-Monovette tubes). After sample collection, plasma was separated within 2 h (3500 rpm for 5 min) and aliquots were stored at –80°C until analysis. Plasma Se was analysed using inductively coupled plasma mass spectrometry (ICP-MS) at the Centre for Trace Element Analysis, Department of Chemistry, University of Otago, Dunedin, New Zealand. C-reactive protein and α_1_-acid glycoprotein were analysed using a Cobas C311 automatic electronic analyser (Roche), in the Department of Human Nutrition Laboratories (University of Otago).

The accuracy and precision of the analyses were checked using certified controls and in-house pooled samples (after every 15 samples), respectively. The analysed mean ± sd (CV) value for the Se control (UTAK Laboratories, Inc.) was 1·32 ± 0·02 μmol/l (1·3 %), compared with the manufacturer’s concentration of 1·37 μmol/l. The mean ± sd (CV) for the C-reactive protein control (Roche Diagnostics) was 9·5 ± 0·4 mg/l (4·6 %), compared with the manufacturer’s concentration of 9·1 mg/l. The multilevel controls for α_1_-acid glycoprotein (Roche Diagnostics) were 0·5 ± 0·01 g/l (1·1 %) and 0·8 ± 0·01 g/l (1·4 %), compared with the manufacturer’s concentrations of 0·7 and 1·2 g/l, respectively.

## Statistical analysis

Plasma Se concentrations were adjusted for inflammation using the Biomarkers Reflecting Inflammation and Nutrition Determinants of Anaemia (BRINDA) regression approach^(25,26)^ calculated for each participant as: Se adjusted = exp (ln Se – (β1 × ln C-reactive protein) – (β2 × ln α_1_-acid glycoprotein)). Differences between groups were estimated using a linear regression model adjusted for parity (1 child *v*. > 1 child) and maternal education (non-tertiary *v*. tertiary).

Usual Se intakes were used to calculate the number of participants with an intake below the estimated average requirement (EAR) of 20 μg/d for 1–3 year olds^(27,28)^. Logistic regression was used to determine whether the odds of having Se intakes below the EAR were different between groups after adjustment for parity and maternal education.

An analysis of the Control group was used to determine the contribution of breast milk, infant formula and food groups towards daily Se intakes. The percentage contribution of each food group to Se intakes for consumers, and for the total sample, was calculated as medians and 25th and 75th percentiles (inter-quartile range). Only the Control group was included in this analysis because the BLISS intervention changed eating behaviour^(13)^.

Univariate unadjusted linear regression analyses were used to describe associations between plasma Se (adjusted for inflammation) and potential predictor variables. These variables were decided *a priori* based on previous associations described in the literature^(7,29–32)^ or were considered to be potentially associated with plasma Se concentrations for mechanistic reasons. Maternal predictor variables were household deprivation, maternal education, maternal age, smoking status during pregnancy; child predictor variables were ethnicity, sex, weight gain and linear growth between 6 and 12 months, plasma Zn, Fe deficiency anaemia, body Fe, intakes of energy, protein, Se, ‘breads and cereals’, ‘meat’, ‘dairy’, and ‘fruits and vegetables’ and consumers of breast milk, infant formula and ‘seafood’. Prior to the regression analysis, smoking status during pregnancy was collapsed into two categories: none (*n* 89) and any (*n* 8; which included daily smokers (*n* 2), occasional smokers (*n* 2) and those who quit during pregnancy (*n* 4)). Plasma Zn concentration was adjusted for time of blood sampling (variable adjusted for reference time of 08.00 hours) and time since last meal (variable adjusted for reference period of 90 min)^(33)^ using the regression equation: Zn time adjusted = exp (ln plasma Zn – (β1 × adj_08.00 hours) – (β2 × adj_90 min)). Following this, an adjustment was also made to plasma Zn concentrations for inflammation using the same approach as for Se explained above. Body Fe was calculated in mg/kg using the equation: –(log10 (soluble transferrin receptor × 1000/plasma ferritin)–2·8229)/0·1207^(34)^ and a body Fe concentration < 0 mg/kg and a Hb concentration < 110 g/l was used to define Fe deficiency anaemia^(15)^. All continuous variables were standardised to allow for comparison of the regression coefficients; the dietary variables were calculated as daily intakes in grams. In the adjusted multivariate regression analysis, variables were chosen to be included in the model if the regression coefficient was ≥ 0·06 μmol/l per unit (this number is approximately 0·3 sd, commonly referred to as a ‘small effect’^(35)^) or with a *P* < 0·1, and were adjusted for infant sex.

All analyses were conducted using Stata, version 15.1 (StataCorp LP). *P* values < 0·05 were considered to be statistically significant.

## Results

A total of 101 participants (*n* 51 Control and *n* 50 BLISS) contributed both dietary intake data by 3-d weighed diet record and a plasma Se sample (Fig. 1) at 12 months of age (50 % of initial sample). Although only twenty-two participants (10·6 %) had formally withdrawn from the study at this stage, a number of participants in both groups did not provide dietary (*n* 11), biochemical (*n* 41) or both (*n* 53) types of data making them ineligible for these analyses. Maternal, household and infant characteristics of those included in this analysis are shown in Table 1. There was no evidence of differences between those included and excluded from this analysis (all *P* > 0·05) except for maternal education (those included were more likely to have a university education: 57 % compared with 40 %, *P* = 0·044) and maternal age (those included were 2·4 years older on average, *P* = 0·002).

**Table 1.** Characteristics of participants who provided dietary intake and plasma Se data at 12 months of age (Numbers and percentages; mean values and standard deviations)

Variables	Control (*n* 51)		BLISS (*n* 50)	
	*n*	%	*n*	%
**Maternal and household**				
Maternal age at birth (years)				
Mean	32·7		32·4	
sd	5·6		4·5	
Maternal parity				
First child	18	35	22	44
Two children	22	43	20	40
Three or more children	11	22	8	16
Maternal ethnicity				
New Zealand European	45	88	39	78
Māori	6	12	5	10
Other	0	0	6	12
Maternal education				
School only	14	27	12	24
Post-secondary	7	14	10	20
University	30	59	28	56
Household deprivation*				
1–3 (low)	14	27	14	28
4–7	25	49	26	52
8–10 (high)	12	24	10	20
**Infant**				
Sex				
Female	24	47	32	64
Male	27	53	18	36
Ethnicity				
New Zealand European	39	77	35	70
Māori	12	23	9	18
Other	0	0	6	12
Infant birth weight (g)†				
Mean	3546		3525	
sd	459		449	
Infant gestational age at birth (weeks)				
Mean	39·8		40·0	
sd	1·5		1·2	

* Household deprivation categorised into: 1–3 (low), 4–7 and 8–10 (high) using the New Zealand Index of Deprivation 2013. The index combines different dimensions of deprivation from New Zealand census data. A deprivation score is assigned to each meshblock (geographical area defined by Statistics New Zealand)^(19)^.

† Available data for Control *n* 50 and BLISS *n* 48.

### Selenium intake and status

Usual Se intakes are shown in Table 2. The OR of Se intakes below the EAR of 20 μg/d^(27,28)^ was no different between the two groups (OR: 0·89; 95 % CI 0·39, 2·03) (Table 2). The highest usual Se intake was 37 μg/d. No dietary supplements containing Se were taken. Plasma Se concentrations (adjusted for inflammation) are also shown in Table 2. Plasma Se concentrations without adjustment for inflammation are shown in online Supplementary Table 1.

**Table 2.** Usual daily Se intake and status of 12-month-old toddlers by complementary feeding approach (Mean values and standard deviations; mean differences and 95 % confidence intervals; odds ratios and 95 % confidence intervals)

	Control (*n* 51)		BLISS (*n* 50)		Mean difference*	95 % CI	OR	95 % CI
	Mean	sd	Mean	sd				
Dietary intake†								
Energy (kJ/d)	3648	658	3510	619	–139	–394, 116		
Protein (g/d)	31·3	7·6	29·7	7·4	–1·7	–4·6, 1·3		
Se (μg/d)	18·2	6·3	19·2	5·8	0·9	–1·5, 3·2		
Below EAR‡, *n* (%)	32	63	30	60			0·89	0·39, 2·03
Biochemical status								
Plasma Se (μmol/l)§	0·81	0·17	0·85	0·18	0·04	–0·03, 0·11		

EAR, estimated average requirement; AGP, α_1_-acid glycoprotein; CRP, C-reactive protein.

* Mean differences and 95 % confidence intervals for BLISS compared with Controls (adjusted for maternal education and parity).

† Intake reported in the 3-d weighed diet records collected at 12 months of age, and usual dietary intakes calculated using the Multiple Source Method^(24)^.

‡ Se intake below the EAR of 20 μg/d for 1–3 year olds^(27,28)^.

§ Adjusted for inflammation using the BRINDA^(25)^ approach: exp (unadjusted ln plasma Se−(regression coefficient for CRP) × (CRP−(maximum of lowest decile for CRP))−(regression coefficient for AGP) × (AGP−(maximum of lowest decile for AGP))).

### Food groups contributing to selenium intakes

The food group contributing the most to total Se intakes of 12-month-old toddlers appeared to be breast milk, contributing 20 % in the whole sample and 39 % when restricted to those who consumed breast milk (Table 3). In total, ‘breads and cereals’ contributed the most Se (12 %), followed by ‘meat’ (11 %) and ‘dairy’ (10 %). When data for ‘consumers’ only were analysed both ‘breads and cereals’ (12 %) and ‘meat’ (12 %) equally contributed to Se intakes. Of those consuming infant formula (the infant formulas consumed in this study contained between 0 and 17·3 μg Se per 100 g), formula intakes contributed 9 % of daily Se intakes (Table 3).

**Table 3.** Contribution of food groups to the Se intakes of toddlers at 12 months of age*,† (Numbers and percentages; median values and interquartile ranges)

	Number of consumers		Contribution of food group to Se intake of consumers‡, %		Contribution of food group to Se intake of total sample (*n* 51), %	
	*n*	%	Median	IQR§	Median	IQR§
Food group						
Breast milk	28	55	39	35–49	20	0–40
Breads and cereals	51	100	12	7–20	12	7–20
Meat||	47	92	12	7–22	11	5–21
Dairy products¶	51	100	10	5–15	10	5–15
Infant formula	24	47	9	0–40	0	0–2
Seafood**	8	16	9	2–21	0	0–0
Fruits and vegetables	51	100	6	4–11	6	4–11
Eggs	31	61	4	2–28	1	0–9
Miscellaneous††	50	98	2	0–4	2	0–4
Nuts and seeds	24	47	1	0·6–2	0	0–1
Legumes	21	41	0·3	0·1–3	0	0–0·3

IQR, inter-quartile range.

* Only Control group included (*n* 51).

† Intake reported in the weighed 3-d diet records collected at 12 months of age.

‡ Ordered by the food group contributing the most to Se intakes by consumers (‘Consumers’: the contribution of Se intakes of the toddlers consuming this food group (e.g. breast milk contributes a median of 39 % of Se intakes for the twenty-eight toddlers consuming breast milk)).

§ Data expressed as median percentages (NB: mean percentages added to 100 % of total Se intakes from food groups).

|| Comprises all meat: red meat, poultry, pork, processed meat, etc.

¶ Includes cows’ milk as a drink.

** Comprises fish and shellfish.

†† Miscellaneous comprises: fats, sugar, sweet foods, herbs and spices, sauces, spreads, beverages, etc.

### Factors associated with plasma selenium concentrations

Univariate and multivariate associations between factors decided *a priori* and plasma Se concentrations (adjusted for inflammation) are shown in Table 4. In the unadjusted analysis, toddlers of mothers who reported any smoking (daily, occasional, quit during) during pregnancy had on average 0·16 μmol/l lower plasma Se concentrations compared with toddlers of mothers who did not smoke during pregnancy, although this association was attenuated in the adjusted model (0·13 μmol/l; 95 % CI −0·25, −0·003). ‘Seafood’ intake in toddlers was associated with higher plasma Se concentrations in the unadjusted analysis (0·12 μmol/l; 95 % CI 0·04, 0·20), but this association was also attenuated in the adjusted analysis. Consuming breast milk was associated with 0·12 μmol/l (95 % CI −0·19, −0·04) lower plasma Se concentrations in toddlers in the adjusted model. The *R* ^2^ for the final multivariate model was 0·30 indicating that 30 % of the variance in plasma Se concentration was explained by the factors included in this model.

**Table 4.** Factors associated with plasma Se concentrations (μmol/l, adjusted for inflammation) in toddlers at 12 months of age (Regression coefficients and 95 % confidence intervals)

	Unadjusted (*n* 101)		Adjusted (*n* 96)*	
	Regression coefficient	95 % CI	Regression coefficient	95 % CI
Household deprivation†				
1–3 (low)	0·00	–0·08, 0·09	0·01	–0·06, 0·09
4–7	Reference		Reference	
8–10 (high)	–0·08	–0·17, 0·00	–0·03	–0·11, 0·06
Ethnicity (child)				
New Zealand European	Reference			
Other	0·02	–0·06, 0·10		
Sex (child), female‡	0·03	–0·04, 0·10	0·04	–0·02, 0·11
Maternal education				
School only	Reference		Reference	
Post-secondary	0·07	–0·04, 0·18	0·01	–0·09, 0·12
University	0·02	–0·06, 0·11	0·01	–0·07, 0·09
Maternal age at infant birth (years)	0·00	–0·01, 0·01		
Smoking status during pregnancy§	–0·16	–0·28, −0·03	–0·13	–0·25, −0·003
Plasma Zn||,¶	0·02	–0·01, 0·06		
Fe deficient anaemia**	–0·09	–0·17, −0·003	–0·08	–0·17, 0·02
Body Fe¶,††	0·02	–0·02, 0·05		
Energy intake¶,‡‡	–0·01	–0·05, 0·02		
Protein intake¶,‡‡	0·02	–0·01, 0·06		
Se intake¶,‡‡	0·04	0·002, 0·07	0·03	–0·01, 0·07
Consumes any breast milk§§	–0·12	–0·19, −0·05	–0·12	–0·19, −0·04
Consumes any infant formula||||	0·03	–0·04, 0·10		
Consumes any ‘seafood’¶¶	0·12	0·04, 0·20	0·08	–0·002, 0·16
‘Bread and cereal’ intake¶	–0·01	–0·04, 0·03		
‘Meat’ intake¶	–0·01	–0·05, 0·02		
‘Dairy’ intake¶	0·02	–0·02, 0·05		
‘Fruit and vegetable’ intake¶	–0·01	–0·05, 0·02		
Weight gain between 6 and 12 months¶,***	0·02	–0·02, 0·06		
Linear growth between 6–12 months¶,***	0·00	–0·04, 0·04		

* Variables chosen to be included in the final model were those with regression coefficient ≥ 0·06 μmol/l or *P* < 0·1, adjusted for child sex.

† Household deprivation categorised into: 1–3 (low), 4–7 and 8–10 (high) using the New Zealand Index of Deprivation 2013. The index combines different dimensions of deprivation from New Zealand census data. A deprivation score is assigned to each meshblock (geographical area defined by Statistics New Zealand)^(19)^.

‡ Female (*n* 56) compared with male (*n* 45).

§ Data available for *n* 97; any smoking (*n* 8) compared with no smoking (*n* 89).

|| Plasma Zn adjusted for time of blood sampling and time since last meal^(33)^ and inflammation^(25)^.

¶ Standardised continuous variable; dietary intake variables as daily intakes in grams.

** Data available for *n* 100; yes (*n* 19) compared with no (*n* 81), Fe deficiency anaemia defined as Hb < 110 g/l and body Fe < 0 mg/kg.

†† Body Fe calculation (mg/kg): –(log10(sTfR × 1000/ferritin)−2·8229)/0·1207 from Cogswell *et al.* ^(34)^

‡‡ Usual intakes: calculated using the Multiple Source Method^(24)^ from 3-d weighed diet records.

§§ Yes (*n* 56) compared with no (*n* 45).

|||| Yes (*n* 42) compared with no (*n* 59).

¶¶ Yes (*n* 24) compared with no (*n* 77).

*** Data available for *n* 97.

## Discussion

The current results demonstrate that amongst New Zealand toddlers (12 months of age) many have Se intakes that are below the EAR, this is regardless of how complementary foods are introduced to them as infants, and there was no difference in plasma Se concentrations between toddlers who had followed a baby-led approach to complementary feeding and those who followed traditional spoon-feeding. Of the infant milk sources (breast milk and infant formula), breast milk appeared to contribute the most to dietary Se intakes of consumers in this age group. For those who consumed them, ‘breads and cereals’ and ‘meat’ contributed the most Se of all the food groups, followed by ‘dairy’. In the adjusted analysis, consumption of breast milk was negatively associated with plasma Se concentrations in toddlers. Maternal smoking during pregnancy had a weak negative association, and consumption of ‘seafood’ had a weak positive association, with plasma Se concentrations.

While very few studies have assessed Se intakes in young New Zealand children, our mean Se intakes of 18·2 μg/d for Control and 19·2 μg/d for BLISS toddlers were both higher than previously reported intakes of 13·7 μg/d for New Zealand toddlers (12–24 months) collected two decades earlier^(7)^. However, the exclusion of breastfed toddlers from the study by McLachlan *et al.* ^(7)^ may contribute to some of the discrepancy here, as well as the fact that the earlier New Zealand Food Composition Database may not have sufficiently estimated regional variations in the Se content of foods. Our results suggest that more than half (63 % Control, 60 % BLISS) of the toddlers in this study had Se intakes below the EAR; however, it has been recently suggested that there is a great need for change to the dietary reference values for Se (for all age groups), given that the recommended intake values were set when there was scarce evidence on the health effects of Se^(36)^.

Se is considered to be highly toxic at the upper level of intake, or UL (usually as a result of high intakes of Se supplements), with reported symptoms of hair, skin and gastrointestinal abnormalities, and fatigue^(37)^. While no toddlers in this study consumed Se supplements, and none had total intakes above the upper level of intake (90 μg/d^(28)^), evidence in adults suggests that long-term exposure at intakes lower than the current adult UL may increase the risk of type-2-diabetes^(38)^ and this has led to debate and a call for an up to date risk assessment on the adverse effects levels for all ages^(39,40)^.

The main (non-infant milk) food group contributing to Se intakes of the total study sample was ‘breads and cereals’, consistent with previous findings in young New Zealand children^(7,31)^. While offal is a good source of Se^(41,42)^, only one participant consumed any (chicken liver pâté) at 12 months of age. The infant formulas consumed had a wide range of Se concentrations (between 0 and 17·3 μg/100 g), but interestingly, none of the ‘toddler milks’ (marketed for toddlers > 12 months of age) contained added Se. The contribution of Se intakes from different infant formulas (with varying Se concentrations) was not assessed in this study.

The finding that breast milk consumption appeared to be the largest contributor to Se intake was surprising given that consuming breast milk was associated with poorer Se status. There are a number of possible explanations for this and this finding should be treated with caution. First, it was not possible to measure breast milk intake, and the single value used for breast milk intake^(23)^, while used in other studies, was not generated using New Zealand data so may not accurately reflect actual breast milk intake in these toddlers. Second, the Se content of breast milk is dependent on the Se status of the mother^(43)^, but we were not able to directly measure the Se concentration of breast milk consumed by toddlers in this study. Instead, a Se value of 2 μg/100 g taken from the New Zealand Food Composition Database^(20)^ was used, which may be too high, especially for South Island mothers and their infants. Clearly, analysis of infant breast milk intake and of the Se concentrations of breast milk from mothers in the South Island of New Zealand is urgently required.

The mean plasma Se concentrations reported in this study were 0·81 μmol/l for Control and 0·85 μmol/l for BLISS infants, which were higher than has been previously reported in infants (0·69 μmol/l), toddlers (0·61 μmol/l) and young children (0·79 μmol/l) in the South Island of New Zealand^(7,31)^. The results are also at the upper end of the range for infants and young children reported in studies internationally: 0·67–0·83 μmol/l^(29,30,44)^, which is surprising given the lower Se concentrations reported in this region. There are currently no universal (age appropriate) criteria for low plasma Se due to the wide variability in the soil Se concentrations of different geographical locations^(42)^, and the paucity of data on possible health effects. Earlier research suggested a cut-off of ≤ 0·82 μmol/l as a level considered to ensure optimal activity of enzymes (iodothyronine 5’ deiodinases) associated with thyroid function^(45)^; however, there is little evidence that concentrations above this cut-off are beneficial to all areas of human health^(36)^. The current study did not assess any health effects related to the plasma Se concentrations reported, and long-term implications of low Se status in this age group remain relatively unclear, as does the specific level/cut-off for defining low plasma concentrations.

For all toddlers, maternal smoking status during pregnancy and breast milk consumption were negatively associated with plasma Se concentrations after adjustment for child sex and other potential predictor variables in the model. Lower Se status of young children has previously been shown to be associated with parental^(29)^ and household smoking^(7)^. Se intakes below recommendations have been reported in the New Zealand population^(6,7)^ and low maternal Se status reduces breast milk Se concentrations^(43)^ which could be contributing to the association between breast milk consumption and toddlers’ plasma Se concentrations.

This is the first study to assess whether the baby-led or spoon-feeding approach to complementary feeding has an impact on Se status in toddlerhood. Rigorous methods were used for the dietary analysis to ensure regional variations in food Se concentrations were taken into account, an approach not possible previously^(31)^, and to adjust the distribution of observed intakes to estimate usual Se intakes. However, our study also has some limitations. This was a secondary analysis of the BLISS study which was not specifically designed or powered to assess Se intake and status. Se intake from breast milk was estimated, because the volume of breast milk and concentration of Se in breast milk were not measured. There were a large number of participants excluded from this analysis because of incomplete data. Lastly, the study was not designed to determine the specific health effects related to the Se intakes and plasma concentrations reported and further studies are needed to determine what, or whether any negative health consequences at these plasma Se concentrations occur.

In conclusion, Se intakes and plasma Se concentrations of 12-month-old New Zealand toddlers were no different between those who had followed a baby-led approach to complementary feeding and those who followed traditional spoon-feeding. However, more than half of toddlers had Se intakes below the EAR. Further research is required to determine whether any negative health consequences at these intakes and status occur.

## Supplementary material

For supplementary material accompanying this paper visit https://doi.org/10.1017/S0007114522002379. click here to view supplementary material
