# Supplementary material for: Selenium intakes and plasma selenium of New Zealand toddlers: secondary analysis of a randomised controlled trial
Source: Br J Nutr. 2022 Jul 27;129(7):1193–201. doi: 10.1017/S0007114522002379 (PMC10011592; doi:10.1017/S0007114522002379)
Supplement: Supplementary file 1 [file S0007114522002379sup001.docx]

**Supplementary Table 1.** Plasma selenium (without adjustment for inflammation) concentrations of BLISS study participants at 12 months of age

|  | **Control (*n*=51)** | **BLISS (*n*=50)** | **Difference (95% CI) *** |
| --- | --- | --- | --- |
| Plasma selenium† (μmol/L), mean (SD) | 0.80 (0.17) | 0.85 (0.19) | 0.03 (-0.04, 0.1) |

* Difference adjusted for infant age and sex, maternal education and parity

† Values were not adjusted for inflammation
